# Automatically identifying the function and intent of posts in underground forums

**DOI:** 10.1186/s40163-018-0094-4

**Published:** 2018-11-29

**Authors:** Andrew Caines, Sergio Pastrana, Alice Hutchings, Paula J. Buttery

**Affiliations:** 1 Natural Language & Information Processing, Department of Computer Science & Technology, University of Cambridge, Cambridge, UK; 2 Cambridge Cybercrime Centre, Department of Computer Science & Technology, University of Cambridge, Cambridge, UK

**Keywords:** Underground forums, Cybercrime, Deviant behaviour, Machine learning, Natural language processing

## Abstract

The automatic classification of posts from hacking-related online forums is of potential value for the understanding of user behaviour in social networks relating to cybercrime. We designed annotation schema to label forum posts for three properties: post type, author intent, and addressee. The post type indicates whether the text is a question, a comment, and so on. The author’s intent in writing the post could be positive, negative, moderating discussion, showing gratitude to another user, etc. The addressee of a post tends to be a general audience (e.g. other forum users) or individual users who have already contributed to a threaded discussion. We manually annotated a sample of posts and returned substantial agreement for post type and addressee, and fair agreement for author intent. We trained rule-based (logical) and machine learning (statistical) classification models to predict these labels automatically, and found that a hybrid logical–statistical model performs best for post type and author intent, whereas a purely statistical model is best for addressee. We discuss potential applications for this data, including the analysis of thread conversations in forum data and the identification of key actors within social networks.

## Introduction

Underground communities attract actors interested in illicit and black hat articles. Concretely, web forums are used for the exchange of knowledge and trading of illegal tools and services, such as malware, services to perform denial-of-service attacks or zero-day exploits. Understanding the social relationships and evolution of actors in these forums is of potential interest to design early intervention approaches or effective countermeasures. However, the analysis of these forums is challenging for various reasons. First, the large volume of data requires automatic tools for extracting knowledge (see an overview of  "Related work" section). Second, the use of non-standard language, including specific jargon and frequent spelling and grammatical errors makes the use of standard language processing tools infeasible.

We present our attempts to automatically classify the function and intent of texts from online hacking-related forums. The overall aim of our work is to better understand the social networks which emerge in online forums. We seek to identify the key actors, which tools and techniques they are talking about, in what way and with whom, and how we can analyse and understand the language they are using. The first step in this project is to optimally extract information from our raw data: the texts from individual forum posts. Since we are working with a corpus containing millions of posts, manual information extraction is infeasible. Instead we aim to automatically classify the data with labels of interest to us. We identify three initial information classes which we would like to extract for each post: the post type—whether it is a question, an offer, and so on; the author’s intent in writing the post; and who the post is addressed to.

In later work we intend to add further information types to our classification model, such as the topic and sentiment of the posts. Here we report on our annotation of a gold-standard subset of the CrimeBB Corpus (Pastrana et al. 2018b) and describe the training and evaluation of machine learning models compared with baseline logical rules. Having expected statistical approaches to work best, we in fact find that for labelling post type and author intent, a hybrid of the logical and statistical models performs best. For labelling post addressee, a purely statistical model works best. We show how the information extracted in this way can be put to use in analysis of forum users, and discuss the challenges which lie ahead including the need to normalise forum texts so that we can better analyse them. Note that we do not propose innovative techniques but rather work with a new dataset on a novel problem, develop a text classifier adapted to the domain while also demonstrating a method to do so which involves manual labour but has high quality results.

## Related work

Various researchers have studied the linguistic and behavioural conventions of online forums, and furthermore the best methods for information retrieval and text mining in this domain. Hoogeveen and colleagues (2018) provide a comprehensive overview of the field of web forum retrieval and text analytics. They divide the set of tasks in two: those relating to retrieval and those relating to classification. Our interests span both task types for the purpose of forum user analysis and classification: here we consider classification within the context of information retrieval. Hoogeveen and colleagues look at many forum types, while we focus on hacking-related forums.

Information retrieval refers to the extraction of content, facts, and relations from collections of text and other media. Classification is a type of machine learning which predicts the most probably label *y* for an instance *X* (in our case a document). Machine learning may generally be *supervised* to some degree by human labelled training data. Unsupervised learning involves a fully automated approach without any pre-labelled training data. Semi-supervised learning relies on a seed set of labelled training instances to start from, with the remainder (usually larger) being unlabelled; the learning algorithm ‘bootstraps’ from that seed set in a process which is often found to improve on fully unsupervised learning. We adopt a supervised approach in which our classifier is trained on human labelled data only, since this type of machine learning is still held to yield the highest accuracy outcomes. However, there is clearly a trade-off between accuracy and the human labour involved in preparing the training data. We opted for a supervised approach since the domain is non-standard, linguistically-speaking, and we wished to fully explore and understand the type of data we are dealing with. In future work, though, semi-supervised approaches may be of use, as we indeed have a much larger corpus of unlabelled texts than we can feasibly annotate in any reasonable amount of time.

Meanwhile Lui and Baldwin (2010) share our interest in categorising forum users, though they do so with a higher dimensional schema than the one we use, labelling the clarity, positivity, effort and proficiency found in users’ forum contributions. Thus they can classify a user as an ‘unintelligible, demon, slacker hack[er]’ (in order of clarity, positivity, effort, proficiency), at worst, or a ‘very clear, jolly, strider guru’ at best. Multiple annotators labelled a reference set on the basis of users’ texts, and automatically extracted features were used in a machine learning experiment. Their features include the presence of emoticons, URLs and ‘newbie’ terms (all Booleans), word counts, question counts, topic relevance and overlap with previous posts in the thread. We use similar features, and can investigate implementation of their full set in future work.

Portnoff and colleagues (2017) aim to identify forum posts relating to product or currency trade, and to determine what is being bought or sold and for what price. This work has many similarities to ours, in that the first task is to classify posts into different types, and identifying the entities being discussed is a subsequent task of interest. However, they only seek to retrieve posts relating to trade, a narrower focus than ours. We concur with their observation that forum texts are not like those found in ‘well-written English text of *The Wall Street Journal*’, and consequently off-the-shelf natural language processing (NLP) tools, such as part-of-speech taggers, syntactic parsers, and named entity recognisers (as might be used to identify products) perform poorly in this domain. In response they discuss NLP ‘building blocks’ which might support human analysis of trade in forum data, essentially using lexico-syntactic pattern matching to good effect for the retrieval of products, prices and currency exchange from online forum texts.

Durrett and colleagues elaborate on the Portnoff et al. paper by discussing forum data in the context of ‘fine-grained domain adaptation’, showing that standard techniques for semi-supervised learning and domain adaptation (e.g. Daumé 2007; Turian et al. 2010; Garrette et al. 2013) work insufficiently well, and that improved methods are needed (Durrett et al. 2017). At the moment we adopt a holistic view of user behaviour on forums; however, if in future work we decide to focus on subsections of forum activity, such as trade-related activity, then the findings and proposals of Portnoff, Durrett and colleagues will be valuable and influential to our own methods.

Li and Chen (2014) construct a pipeline of keyword extraction, thread classification, and deep learning based sentiment analysis to identify the top sellers of credit card fraud techniques and stolen data. All stages of their pipeline are of relevance to us because the ‘snowball sampling’ (a.k.a ‘bootstrapping’) method they use for keyword extraction is one we could employ in future work to accelerate knowledge discovery. Thread classification is one of the tasks we discuss in this report, as is sentiment analysis, while ‘deep learning’ (i.e. unsupervised machine learning with neural networks) is a technique of great potential for the type and size of data we are working with. In Li and Chen’s work, sentiment analysis is used as it is so often used—to assess whether people have reviewed a product positively or negatively—but what is unusual here is that, rather than, say, Amazon, the reviewing forum is a blackhat site, and rather than books, toys or other general consumer goods, the product under review has criminal intent or has been illegally obtained. This is a noteworthy revision of ‘vanilla’ sentiment analysis and one we can consider for future research using the CrimeBB dataset.

Our work therefore builds on the work of others in the field by adopting existing information retrieval and text classification approaches, applying them to a corpus of wider scope than previously used, and using the resultant dataset for downstream analysis of social networks and identification of key actors in cybercrime communities.

## The CrimeBB Corpus

We work with sections of CrimeBB, a collection of posts from online English and Russian-language forums collected using the CrimeBot tool described in Pastrana et al. (2018b). The corpus is updated regularly and therefore continues to grow; at the time of writing, CrimeBB numbered 48.3 million posts from 0.9 million contributors to 1005 bulletin boards (Table 1).

**Table 1:** Contents of the CrimeBB Corpus

Forum	Boards	Members	Threads	Posts	Oldest post
HackForums	175	557,406	3,789,274	39,448,526	01-2007
Kernelmode	16	1430	3091	24,885	03-2010
Offensive Community	63	9786	11,460	49,426	06-2012
Multiplayer Game Hacking	699	375,989	729,565	8,798,105	12-2005
Stresserforums	22	573	568	5308	04-2017
Greysec	30	430	1231	6923	06-2015
Total	1005	945,614	4,535,189	48,333,173	12-2005

HackForums is the largest forum included in CrimeBB; the oldest HackForums posts were made more than a decade ago, and it has long been ranked as the top hacking forum by Alexa.com. HackForums has been linked to several high profile events relating to denial of service attacks and banking malware. The other five forums are smaller in size compared to HackForums. However, one (Multiplayer Game Hacking) is older, dating back to 2005.

In this paper we analyse subsets of CrimeBB from a number of bulletin boards^1^ on HackForums, including Beginners Hacking, Premium Sellers, and Remote Administration Tools. These were chosen as a representative sample of board types found on forum sites. In total we extracted 2200 posts and annotated them as described below, before training machine algorithms to scale up the annotation process.

### Annotation

To begin to understand our data and the challenges contained therein we selected a sample of posts from HackForums and annotated each one for variables of interest. We selected 1000 posts from the Beginners Hacking bulletin board, 200 posts from Premium Sellers, and another 1000 posts from thirteen randomly chosen boards (‘mixed boards’).^2^ The selection process involved randomly sampling from the history of the chosen bulletin boards, filtering for threads with fewer than 20 posts (so that our dataset would not be dominated by a few lengthy threads) until we reached the required number of posts. We then annotated the posts for three variables: post type, author intent and addressee. These chosen variables reflect our interest in identifying *who* is saying *what* to *whom*.

*Post type* indicates the general function of the post and can take one of seven labels, as shown in Table 2 with illustrative examples. An **offerX** and a **requestX** involve products and services and are opposites of each other; similarly, we see **exchange** posts when a user proposes the trade of a product and/or service for a currency amount or another product and/or service in kind.

**Table 2:** Post type labels in the CrimeBB Corpus, with anonymised examples from HackForums

Post type	Description	Anonymised example
OfferX	The user offers a product or service	I am looking to sell this domain domain_name
RequestX	The user requests a product or service	Looking for a product on here
Exchange	The user proposes a trade	I will buy it for cash_value
Tutorial	The user points to a tutorial	...here’s the steps url
Info request	The user requests information	What’s the estimated release of the api
Comment	The user comments on a discussion	product need to be portforwarded
Social	The user attempts to connect with another user for social reasons	If anyone social_activity then post here)

Informational post types include **tutorials** in which a link or guide showing how to perform a certain task is posted for others to follow. An **info request** is a request for help, guidance or information, often being the first post in a thread conversation. A **comment** is any response which is simply informative, whether it is the first post in a thread, or a response to the post(s) of others. Finally, a **social** post is one in which a user proposes subsequent interaction, perhaps through another medium such as networked gaming.

**Table 3:** Author intent labels in the CrimeBB Corpus, with anonymised examples from HackForums

Author intent	Description	Anonymised example
Positive	Posts which are helpful, give praise or encouragement, express amusement or a desire for a product/service	If service is available just follow the same procedure; nice design BTW
Neutral	Posts which have no discernible sentiment, positive or negative	There’s currently number members registered
Negative	Posts that state a negative opinion	I hate product not gonna use it
Arbitrate	Posts containing admin-esque attempts to control discussion	If it’s a person, do not charge
Vouch	Posts that give a positive review of someone’s product/service	Vouch for this person
Gratitude	Posts that thank the authors of previous posts for their contributions	ty ty hope u enjoy
Private message	Posts where the author suggests that communication continues through another channel such as Skype or MSN	Going to messaging-service you now
Aggression	Posts that are abusive to the previous discussion participant(s) in some way	What the fuck are you talking about?

*Author intent* relates to author affect and what they seem to have intended by the specific wording of their posting (Table 3). We propose eight author intent labels^3^ including **positive**, **negative** and **neutral** which are a conventional trio of labels in sentiment analysis—the NLP field this task most closely resembles (e.g. Pang et al. 2002).

Additional author intent labels include **arbitrate**—when a user indicates that a previous post does not belong in the present bulletin board or forum, for some reason; **vouch** and **gratitude** when a user indicates that a product or service is genuine and performant (vouch) or when they wish to thank another user for their help or services (gratitude). Note that these are both sub-types of positive intent, though more specific and serving functions which are important to the maintenance of social relations in an online hacking forum. Also there is **private-message** for when a user attempts to move conversation to another channel such as Skype or MSN; and finally **aggression** which involves abusive language and a hostile stance from one user towards another. Again we have a sub-type of an over-arching label—in this case ‘negative’—as we are interested in the development of hostility in forum interaction.

Finally, we labelled the addressee of each post, which is not always a straightforward task. When there is no obvious individual addressee we reverted to a generic group label such as, ‘thread’ (participants in the discussion so far) or ‘bulletin board’ (all users attending to the bulletin board on which the thread is posted).

Note that each post can be multi-purpose or addressed to more than one interlocutor; therefore we allow multi-labels for all annotation categories. As a final point we emphasise that these annotation categories and labels were deemed suitable for the HF2200 subset. We welcome additions or amendments to our framework, if others deem it advisable having inspected further sections of CrimeBB.

### Annotation agreement

We show inter-annotator agreement for each labelling task and each dataset in Table 4, reporting pairwise proportional overlap between annotators, and Fleiss’s kappa across all three annotators. Fleiss’s $\kappa$ is a measure of agreement between multiple annotators on a fixed set of items using categorical labels (whereas Cohen’s $\kappa$, for instance, assesses agreement between two raters only). It indicates the degree of agreement above chance and is a generalisation of Scott’s $\pi$ rather than Cohen’s $\kappa$ Fleiss (1971). Landis and Koch (1977) proposed that $\kappa$ greater than 0.2 may be interpreted as ‘fair agreement’ (above ‘poor’ or ‘slight agreement’ for less than 0.2), $\kappa$ greater than 0.4 indicates moderate agreement, 0.6 points to substantial agreement, and above 0.8 indicates ‘almost perfect agreement’.

**Table 4:** Inter-annotator agreement for the labelling of HF2200 posts by three annotators

Bulletin board	Task	Annotator 1&2	Annotator 1&3	Annotator 2&3	Fleiss’s $\kappa$	Agreement
Beginners hacking	Post type	.917	.908	.928	.736	Substantial
	Author intent	.760	.535	.537	.239	Fair
	Addressee	.893	.871	.903	.879	Almost perfect
Premium sellers	Post type	.854	.935	.844	.583	Moderate
	Author intent	.849	.834	.799	.405	Moderate
	Addressee	.879	.804	.910	.840	Almost perfect
Mixed forums	Post type	.889	.910	.912	.719	Substantial
	Author intent	.770	.786	.731	.280	Fair
	Addressee	.868	.721	.765	.760	Substantial

Note that the annotation sub-tasks vary in difficulty: post type is fairly straightforward in that it involves relatively clear-cut categories and the type of posting is usually apparent—because it needs to be, else it is questioned (or criticised) by others. Therefore agreement scores tend to be highest for post type labelling, as seen in Table 4. Pairwise inter-annotator agreement is generally good (greater than 70%), albeit lower for author intent than for addressee and post type.

In contrast, the addressee is usually clear but in some cases can be ambiguous or a rhetorical comment to no one in particular. Note also that the majority of posts are addressed to a general audience and that is the source of much of the inter-annotator agreement. The most difficult task of all is discerning the author’s intent, as we inevitably interpret others’ language in idiosyncratic ways, and sometimes users disguise their true intent through sarcasm, or misrepresent their meaning through error or obfuscation. Therefore the assigned labels for author intent are necessarily inferential, sometimes a matter of guesswork, and cannot always be thought of as the user’s true intent (to obtain which we would have to query each author of every post). However, our annotations are a representation of people’s interpretation of the posts, and therefore a proxy for ground truth.

### Annotation counts

We show proportions for each class label in our 2200 annotated posts (HF2200). Since each post was labelled by three annotators, and moreover could have multiple labels, the total number of post type labels in our sample set is $|L_t|=6751$, for author intent it is $|L_i|=7476$, and for addressee, $|L_a|=6650$. In Table 5 we show the frequency of each of our seven post type labels $l_t \in L_t$ as counts and proportions of $|L_t|$, and each of our eight author intent labels $l_i \in L_i$ as counts and proportions of $|L_i|$. With 614 unique addressee labels in HF2200, because of all the individual user IDs, we sort the addressees into three groups—the original poster (OP) of the given thread discussion, some other individual, or a general audience (anyone attending to the bulletin board in question, or all previous participants in a thread).

**Table 5:** Label counts and proportions in HF2200 for each annotation type

Label	Count	Proportion
*Post type*	6751	1
Comment	4821	.714
Info request	1318	.195
OfferX	383	.057
RequestX	136	.020
Tutorial	44	.007
Social	32	.005
Exchange	17	.003
*Author intent*	7476	1
Neutral	3679	.492
Positive	2006	.268
Negative	741	.099
Gratitude	326	.044
Private-message	298	.040
Vouch	217	.029
Arbitrate	134	.018
Aggression	75	.010
*Addressee*	6650	1
Thread OP	3165	.476
General audience	2215	.333
Other individual	1270	.191

## Method and results

With 48.3 million posts in CrimeBB at the time of writing, and the prospect of continuing expansion, it is evidently infeasible to annotate the entirety of the corpus in the way described above. Therefore we turn to machine classification of unseen posts, and in this section present various approaches to the automated labelling of post type, author intent and addressee based on HF2200 as a training set. In the end we find that post type and author intent are best-served by a hybrid logical–statistical approach, while addressee can be most accurately predicted through a statistical model. We note that we may be able to switch to statistical models all round through additional data collection and processing, as well as further feature and algorithm selection experiments in future work.

### Evaluation

We report the performance of our classification models using conventional information retrieval metrics: precision, recall and *F*-measure. To define precision and recall we need counts of true positives (TP), false positives (FP) and false negatives (FN), which are calculated through the interplay of a system’s hypotheses and the ground truth. If for any given post the hypothesised label is the one found in the ground truth, it is a TP; if it is not found in the ground truth it is an FP. In contrast, if a label in the ground truth is not hypothesised, it is an FN. As shown in (1), precision is the count of TPs divided by the total hypothesised labels ($TP+FP$). As shown in (2), recall is the count of TPs divided by the total ground truth labels ($TP+FN$).

1 $$\begin{aligned} Precision=\, & {} \frac{TP}{TP + FP} \end{aligned}$$

2 $$\begin{aligned} Recall=\, & {} \frac{TP}{TP + FN} \end{aligned}$$

Having seen that, as might reasonably be expected, the annotators did not wholly agree in their labels for HF2200 (Table 4), and moreover that annotators were allowed to apply multiple labels to each post for any category, as a consequence there is not always a single ground truth label for post type, author intent and addressee for every post. Often there is, but sometimes not. Indeed we find the multiple labelling allowed in the annotation process informative in itself, and wish to retain this information during classification and evaluation.

Our ground truth label set therefore includes *all* labels found in HF2200. Hence the count of ground truth labels is allowed to be greater than the number of posts in HF2200 (see Table 5 for evidence of this). This detail affects precision and recall in the sense that the count of true positives includes any positive match between the hypothesis and the set of ground truth labels for that post, rather than requiring a full match with the whole set. However, because the size of the ground truth set may be greater than the size of the hypothesis set, and we usually only predict one label per post, the actual maximum recall attainable is less than one (it is the number of predictions over the number of labels in HF2200). One exception is author intent in which we allow the option to add a private-message label to one other prediction, thus increasing the number of predictions to more than one per post. This is a method we can extend through further heuristics, or we can employ a multi-label classifier in future work.

Finally, as is standard, the *F*-measure is the harmonic mean of precision and recall as shown in (3):

3 $$\begin{aligned} F = 2\cdot \left( \frac{Precision \cdot Recall}{Precision + Recall}\right) \end{aligned}$$

All evaluations are carried out using the hand-annotated HF2200 dataset. For the logical models we report performance on HF2200 as a whole; for statistical and hybrid models we report average performance over tenfold cross-validation on the HF2200 set.

### Logical models

Due to the skewed label distributions within classes we can adopt an initial approach of simply choosing the most frequent label for each of the annotation types and treat this as our first baseline method (B1). That is, we propose that every post type is a comment, the author’s intent is neutral, and the addressee is the thread OP, based on the label frequencies shown in Table 5. We find that this is a fairly successful approach, with an *F*-measure of 0.731 for post type as comment, achieved more through precision than recall, 0.587 for neutral author intent again with higher precision than recall, and 0.481 for addressee, this time with much lower precision than the other two classes (Table 6).

**Table 6:** Classification of post type, author intent and addressee in the HF2200 dataset: baseline performance of logical models (tenfold cross-validation; precision, recall, *F*-measure)

Model	Post type			Author intent			Addressee		
	P	R	F	P	R	F	P	R	F
B1: Select most frequent label	.796	.676	.731	.788	.468	.587	.521	.447	.481
B2: Decision lists	.898	.763	.825	.783	.498	.609	.873	.748	.806

These results reflect the nature of the HF2200 posts, with the majority being comments (hence high precision), but this label alone not reflecting the remaining structure in the dataset (hence low recall). Again, the majority of posts have a neutral intent (high precision) and many posts are multiply labelled with neutral and some other label(s)—evidenced by the higher recall than for post type. As for addressee, we will achieve about 50:50 precision if we assert that every post has been addressed to the OP of the thread; however, there are some obvious ways we can qualify our initial baseline, since we can for instance rule that if the thread OP posts again later in the same thread, the addressee cannot be the thread OP (themselves) but must be someone else.

#### Post type labelling heuristics

The previous point brings us on to our next baseline approach: introducing a number of heuristics for post classification, based on our observations of behavioural conventions in HackForums bulletin boards. With this approach we implemented a decision rule classifier and present it as our second baseline model (B2). Our decision rules for post type are listed below:

- IF first post in thread AND
  - IF bulletin board title contains /trading|sellers|bazaar|market/ $\Rightarrow$ PREDICT ‘offerX’
  - ELSE $\Rightarrow$ PREDICT ‘info request’
- ELSE IF question mark in post $\Rightarrow$ PREDICT ‘info request’
- ELSE $\Rightarrow$ PREDICT ‘comment’

These post type labelling rules reflect our empirical observations that the opening post in a thread will be the offer of a product or service if the bulletin board relates to trading—otherwise it is usually an information request—and that information requests in any other thread position are formed by questions with appropriate punctuation (the question mark). Again, as a default we fall back on the most frequent post type, the comment. This approach results in 34% of the label predictions shifting away from ‘comment’ to ‘info request’ and ‘offerX’—yielding a 10.2% improvement in precision and a 5.7% improvement in recall (Table 6).

However, note that we have no baseline heuristics for labelling four of our seven post type labels: requestX, tutorial, social and exchange. This is because attempts to do so led to performance deterioration rather than improvement. Note also that they are the four minority labels in our set; hence we leave these labels as a target for statistical models in the hope that there are regularities in the data we have not detected.

#### Author intent labelling heuristics

For author intent we propose the following decision rules:

- IF first post in thread $\Rightarrow$ PREDICT ‘neutral’
- ELSE IF post contains /vouch/ $\Rightarrow$ PREDICT ‘vouch’
- ELSE IF arbitrate marker in post $\Rightarrow$ PREDICT ‘arbitrate’
- ELSE IF aggression marker in post $\Rightarrow$ PREDICT ‘aggression’
- ELSE IF gratitude marker in post $\Rightarrow$ PREDICT ‘gratitude’
- ELSE IF negative marker in post $\Rightarrow$ PREDICT ‘negative’
- ELSE IF positive marker in post $\Rightarrow$ PREDICT ‘positive’
- ELSE $\Rightarrow$ PREDICT ‘neutral’
- IF private-message marker in post $\Rightarrow$ APPEND ‘private-message’

Again, these rules are based on observations in the data, and implemented with regular expressions for each label marker as follows:

- arbitrate markers = /(violates|against)\s+\w+\s+rules|wrong (section|forum)| can.*t post that|allowed here|t allowed|off(-| )topic|close this thread/;
- aggression markers = /retarded|idiot|you moron|this shit|skid|what the fuck| wtf/;
- gratitude markers = /thank(s|\s+y*o*u|cheers ma)/;
- private-message markers = /\b(pm.*e*d*)\b|\b(hmu)\b|contact me\b|skype| discord/;
- negative markers = /gonna stop|please stop|this is bad|tell me you didn.*t| stopped reading|dubious|stolen|kidding me|gonna vomit|sucks balls|dwc| smilies\/(sad|confused)|:\(/;
- positive markers = /haha|jaja|lo+l|lmao|glws|dope|check out|you (can|should) try|this is great|smilies\/(roflmao|victoire|smile|tongue|haha)|:D/

Note that the final rule adds a ‘private-message’ label (PM) to the intent label already there. We propose this rule on the grounds that for many posts involving PM requests, there was often a multi-label annotation, with a secondary intent (say, positive + PM, or gratitude + PM, and so on). A consequence of this PM rule is to increase the number of predictions (and thus the denominator for the precision calculation) by 7.2%. On the other hand, it is the first time we attempt to mimic the multiple labelling allowed during annotation, and therefore a move towards a more authentic model.

Having applied our set of decision rules for author intent, 30% of predictions are now a label other than the most frequent selection (neutral). Most of the shift is toward positive, private-message, gratitude and vouch labels. As a consequence there is a small deterioration in precision (by 0.5%) but a 3% improvement in recall, leading to a higher *F* score than B1 (Table 6). The impact of introducing the full set of labels to our author intent predictions reflects our finding from the annotation exercise—that author intent is a difficult annotation type to agree on (Table 4)—hence it is no surprise that precision deteriorates for this class once we attempt to go beyond a homogenous most-frequent-label approach.

#### Addressee labelling heuristics

For addressee we have the following decision rules:

- IF first post in thread $\Rightarrow$ PREDICT ‘general audience’
- ELSE IF post contains citation AND
  - IF cited user IS thread OP $\Rightarrow$ PREDICT ‘thread OP’
  - ELSE $\Rightarrow$ PREDICT ‘other individual’
- ELSE IF second or third post in thread AND
  - IF author of post $_{n-1}$ is thread OP $\Rightarrow$ PREDICT ‘thread OP’
  - ELSE $\Rightarrow$ PREDICT ‘other individual’
- ELSE IF post author is thread OP $\Rightarrow$ PREDICT ‘general audience’
- ELSE $\Rightarrow$ PREDICT ‘thread OP’

These new rules result in a 51.6% shift away from the most frequent label (thread OP) and a notable performance improvement: precision increases by 35%, recall by 30%, with an *F*-measure of .806 rather than .481 as it was for B1 (Table 6).

We note that precision is relatively high for all annotation types, indicating that our baseline methods are fairly sensible foundations to build on: they are reasonably accurate in what they attempt to do. However, the generally low recall—especially for author intent—indicates that we are not reaching many of the labels our annotations indicate we should be. At this point we turn to statistical models to improve this aspect of classification performance.

### Statistical models

Our baseline logical approaches achieved reasonable levels of precision (Table 6), especially in the context of variable agreement rates between human annotators (Table 4). One problem of our baseline approach is the relatively low level of recall across the board, acutely so for author intent. Evidently our heuristics do not reach a large proportion of ground truth labels—indeed for post type we did not attempt to do so for several labels, and the presence of multiply labelled posts in our reference set but mainly single-label predictions naturally has a detrimental effect on recall. In order to improve our reach across each label set, we investigate the performance of statistical models in this section.

For all models described in this section, we take a number of pre-processing steps common to natural language processing and information retrieval tasks. Firstly we convert the posts in HF2200 to a document-term matrix—that is, a matrix of counts with the words occurring in HF2200 as column values, and each of the 2200 posts as a row. We convert all posts to lower case characters, ignore numbers, exclude stop words and those words occurring once only (so-called ‘hapax legomena’) as well as words with zero or near-zero variance. These steps shrink our vocabulary from 9533 to 4834 words for the post type dataset, 7286 for author intent, and 4561 for addressee (variance is partly dependent on the distribution of labels). The matrix is then populated with occurrence counts for each word in the vocabulary within each post.

These word counts are then transformed using TF-IDF (‘term frequency $\cdot$ inverse document frequency’), a weighting which promotes words occurring fairly frequently in few documents above those occurring highly frequently but ubiquitously across the corpus (Spärck-Jones 1972). This gives us a vector of weighted word frequencies for each post in HF2200, which we can use as lexical features in classifier training. We also add the following metadata features: post contains an image, post contains a link, post contains code, post contains an iFrame, post formally cites another post, post addressed to the thread’s original post author (OP), post is first post in thread, post author is thread OP, cited post is the first post in thread (all Boolean), bulletin board ID, ordinal of post within its thread. These features were selected as they encode many of the intuitions represented in our decision list classifiers (B2).

We begin with a support vector machine model (SVM) as SVMs are known to work well for text classification tasks, in that they are robust to high-dimensionality and sparse document-term matrices, plus we can trial different types of separator (linear, polynomial, radial basis function, etc) (Joachims 1998). Indeed we tested linear, polynomial and RBF kernels, along with a matrix of cost and loss values, and found that an $\ell _2$ regularised SVM (dual) with linear kernel gave the best performance for our three label types in HF2200. In Table 7 we report mean precision, recall and *F*-measure values for tenfold cross-validation of this statistical model (S1), implemented with the LiblineaR R wrapper for the LIBLINEAR C/C++ machine learning library (Helleputte 2017).

**Table 7:** Classification of post type, author intent and addressee in the HF2200 dataset: performance of statistical models (tenfold cross-validation accuracies; mean precision, recall, *F*-measure)

Model	Post type			Author intent			Addressee		
	P	R	F	P	R	F	P	R	F
S1: Support vector machine	.753	.744	.749	.414	.394	.404	.820	.810	.815
S2: Extreme gradient boosting	.699	.690	.695	.458	.435	.446	.609	.601	.605
S3: Linear model	.760	.751	.755	.492	.467	.479	.819	.809	.814

Our next statistical model is XGBoost: ‘extreme gradient boosting’, a parallel tree boosting algorithm known to be fast and accurate^4^ (Chen et al. 2018). Boosting is an additive technique whereby new models are added to correct the errors made by existing models thus far: models are added sequentially until no further improvements can be made. In gradient boosting, new models predict the residuals or errors of prior models using a gradient descent algorithm. XGBoost is also known to work well with sparse matrices, which is the kind of input associated with textual data. We trained an XGBoost model for each of our three annotation types in HF2200: we set the maximum tree depth at six levels, the number of rounds at 10 and early stopping set to 5, gamma at 1, the learning rate at 0.3, and log loss as our evaluation metric. These settings are fairly conservative, as we wished to avoid over-fitting. The performance of XGBoost is reported in Table 7 as S2. It is apparent that, though fast, XGBoost is not as accurate as SVMs for our given annotation types on this dataset.

Our third and final statistical model is a linear model, trained using LiblineaR (Helleputte 2017). Linear models are well-suited to multi-class classification (as we have here) and LiblineaR is particularly fast compared to other libraries.^5^ We empirically searched for the optimal classification type from seven valid options in LiblineaR, with an optimal cost function, settling on an $\ell _2$ regularised $\ell _2$ loss support vector classification (primal) for post type and addressee, an $\ell _1$ regularised $\ell _2$ loss support vector classification for author intent, and a cost of .001 for post type and author intent, and 1 for addressee. The performance of our linear classification model is reported in Table 7 as our third statistical model (S3).

It is apparent that different statistical model perform best for different annotation types. We propose that for post type and author intent, performance is not overwhelmingly good enough to completely discard the heuristics from our logical models—indeed the baseline decision lists (B2) outperform the statistical models for these annotation types—one problem being that too many predictions are shifted back to the label most frequently found in training, the B1 mode in other words. We see this in a confusion matrix for post type for S3 (linear model) with ‘comment’ (Table 8), and indeed the S3 author intent model, though outperforming S2, simply predicts neutral intent; therefore we prefer to work with S2 (XGBoost) since its performance is not much worse and it does predict intent types other than neutral (Table 9).

**Table 8:** Classification of post type in the HF2200 dataset: linear model (S3) confusion matrix

Prediction	Reference							
	Info request	Comment	OfferX	RequestX	Tutorial	Social	Exchange	Total
Info request	233	43	58	42	4	8	3	391
Comment	311	1700	78	37	21	8	11	2166
OfferX	0	4	29	1	0	1	0	35
RequestX	0	0	0	0	0	0	0	0
Tutorial	0	18	0	0	0	0	0	18
Social	0	0	0	0	0	0	0	0
Exchange	0	0	0	0	0	0	0	0
Total	544	1765	165	80	25	17	14	2610

**Table 9:** Classification of author intent in the HF2200 dataset: XGBoost (S2) confusion matrix

Prediction	Reference								
	Neutral	Negative	Positive	Arbitrate	Aggression	Private-message	Gratitude	Vouch	Total
Neutral	1504	408	955	64	46	124	83	38	3222
Negative	4	0	3	0	0	0	0	0	7
Positive	142	13	28	7	3	5	3	12	213
Arbitrate	8	2	8	3	0	1	0	1	23
Aggression	0	1	1	0	0	0	0	0	2
Private-message	25	1	7	1	0	7	0	1	42
Gratitude	51	4	29	1	0	5	53	5	148
Vouch	14	3	30	1	0	0	1	33	82
Total	1748	432	1061	77	49	142	140	90	3739

Addressee S3 does outperform decision list B2, though, and therefore we retain it as our preferred model. The confusion matrix shows that where there are false predictions, these are most often ‘general audience’, which is not a harmful error because to some extent it is always true, and thus the model acts as we would like it to (Table 10).

**Table 10:** Classification of addressee in the HF2200 dataset: SVM (S1) confusion matrix

Prediction	Reference			
	General audience	Thread OP	Other individual	Total
General audience	675	83	128	886
Thread OP	117	1059	21	1197
Other individual	129	14	362	505
Total	921	1156	511	2588

The one way the addressee model can be thought of as hybrid is in action: when we come to label new data we will continue to use the statistical model for a first pass, and then depending on the predicted label will attempt to identify which individual is addressed (where applicable), who is the thread OP where this is applicable (a trivial task), or whether the post is addressed to participants in the thread or the whole bulletin board where ‘general audience’ is predicted. Thus in processed data there will be an addressee type—one of the three labels presented here—and a more fine-grained addressee label with a user ID or otherwise.

As a note for future work, we can attempt at least another type of statistical model for the prediction of post type, author intent and addressee: neural networks. These are known to have transformed the machine learning field in the past decade and now give state-of-the-art performance for many tasks. We would need to expand our training dataset, since neural networks are known to perform better with many thousands if not millions of instances, whereas our current HF2200 training set is likely to be inadequate. We can investigate accelerated expansion of our labelled data set through crowdsourcing methods, or semi-supervised or unsupervised training methods.

### Hybrid logical–statistical models

The statistical models did not outperform the decision list baseline (B2) for the post type and author intent label types, though did show better recall. Therefore we propose a hybrid approach in order to retain B2’s high precision while addressing its generally low recall through the addition of probabilistic prediction: thus we introduce a statistical model into the decision rules at an appropriate point. For post type the new algorithm is as follows, where argmax() returns the most likely of the defined label-set for the given post:

- IF first post in thread AND IF bulletin board title contains /trading|sellers| bazaar|market/ $\Rightarrow$ PREDICT ‘offerX’
- ELSE PREDICT $\Rightarrow$ argmax(post_type)

That is, instead of falling back on ‘comment’ as our final decision rule, as we did in B2, we train a linear classification model based on all available post type labels—therefore including the four we did not attempt to predict in B2 (‘requestX’, ‘tutorial’, ‘social’, ‘exchange’). This approach yields improved performance as shown in Table 11, outperforming the .898 precision, .763 recall and .825 *F*-measure seen for B2 (Table 6). We also present a new confusion matrix, showing that the majority of predictions continue to be ‘comment’ (which isn’t an egregious error, where it is incorrect), there are many fewer ‘info request’ predictions, and there are several predictions of ‘requestX’ and ‘social’ but still none for ‘tutorial’ or ‘exchange’ (Table 12). It is clear that we need more training examples or improved rules for these post types.

**Table 11:** Classification of post type and author intent in the HF2200 dataset: performance of hybrid models (tenfold cross-validation accuracies; precision, recall, *F*-measure)

Model	Post type			Author intent		
	P	R	F	P	R	F
H1: Logical–statistical hybrid	.919	.781	.844	.786	.499	.611

**Table 12:** Classification of post type in the HF2200 dataset: H1 confusion matrix

Prediction	Reference							
	Info request	Comment	OfferX	RequestX	Tutorial	Social	Exchange	Total
Info request	247	46	16	40	12	7	3	371
Comment	293	1699	58	36	12	8	11	2117
OfferX	4	19	91	3	1	1	0	119
RequestX	0	1	0	1	0	0	0	2
Tutorial	0	0	0	0	0	0	0	0
Social	0	0	0	0	0	1	0	1
Exchange	0	0	0	0	0	0	0	0
Total	544	1765	165	80	25	17	14	2610

For author intent we amend our set of decision rules with a probabilistic prediction if no keywords have been matched, rather than falling back on ‘neutral’ as a default as we did in B2. This step was taken to address the problem found with B2 whereby many negative and positive posts were mis-labelled as neutral. We see from the confusion matrix for this hybrid approach that indeed the predictive model improves the situation to some extent (Table 13). However, it can also be seen that many posts continue to be incorrectly predicted as ‘neutral’, and we presume that this is because it is the dominant label found in the HF2200 dataset (recall Table 5). In future work we can seek to resolve this by taking the number of neutral examples found in any new annotation exercise and placing a bottleneck on how many can be added to the training data for any new statistical model.

- IF first post in thread $\Rightarrow$ PREDICT ‘neutral’
- ELSE IF post contains /vouch/ $\Rightarrow$ PREDICT ‘vouch’
- ELSE IF arbitrate marker in post $\Rightarrow$ PREDICT ‘arbitrate’
- ELSE IF aggression marker in post $\Rightarrow$ PREDICT ‘aggression’
- ELSE IF gratitude marker in post $\Rightarrow$ PREDICT ‘gratitude’
- ELSE IF negative marker in post $\Rightarrow$ PREDICT ‘negative’
- ELSE IF positive marker in post $\Rightarrow$ PREDICT ‘positive’
- ELSE PREDICT $\Rightarrow$ argmax(author_intent)
- IF private-message marker in post $\Rightarrow$ APPEND ‘private-message’

Recall that for addressee, the linear model (S3) outperformed the baseline decision list classifier (B2) and therefore we have no need for a hybrid model, except where it comes to identifying who the individual addressee is, or what type of general audience is involved, as described above.

**Table 13:** Classification of author intent in the HF2200 dataset: H1 confusion matrix

Prediction	Reference								
	Neutral	Negative	Positive	Arbitrate	Aggression	Private-message	Gratitude	Vouch	Total
Neutral	1504	324	807	45	22	107	13	26	2848
Negative	13	16	9	1	0	0	0	1	40
Positive	93	33	130	2	3	6	12	7	286
Arbitrate	9	12	9	25	0	1	0	1	57
Aggression	20	29	15	2	23	0	0	0	89
Private-message	114	31	89	5	7	20	12	12	290
Gratitude	68	9	33	2	1	11	97	3	224
Vouch	34	8	52	0	0	6	18	52	170
Total	1855	462	1144	82	56	151	152	102	4004

This hybrid approach may not suit everyone, since the logical heuristics were naturally time-consuming to develop as they came from the annotators’ observations and generalisations after labelling the sample of 2200 HackForums posts. Indeed the approach is restricted to this domain (that of HackForums for now, but perhaps we can demonstrate that they apply to online hacking forums generally in future work) and therefore needs to be revised for cross-domain transfer. However, the human effort required to label the set of 2200 posts and develop the logical models can be measured in days rather than weeks, and we maintain that there is no better way to understand your data. However, as we show here, the statistical models are not awful on their own, and therefore a purely statistical approach (without a logical hybrid) works well in itself.

## Discussion

We have evaluated machine learning and natural language processing techniques to classify texts from online hacking-related forums. We designed annotation schema to label CrimeBB posts for three properties: post type, author intent, and addressee. Three annotators labelled 2200 posts selected from a range of HackForums bulletin boards, with substantial inter-annotator agreement for post type and addressee, and fair agreement for author intent. To scale up the annotation process, we evaluated the performance of automated approaches to the automatic labelling of posts for post type, author intent and addressee. The best performing set-up is a hybrid logical–statistical classifier for post type and author intent, and a statistical classifier for addressee.

These classification models allow us to quickly label large numbers of posts—the 48.3 million contained in CrimeBB for example, and the thousands of new posts produced each day if we were to implement a daily update pipeline. The labelled posts in turn enable us to analyse the interactions contained in threads and user behaviour across web forums as a whole. For instance, we can identify the creator of a thread, their purpose in doing so (e.g. a request for information, a product for sale, the proposal of a currency exchange), who responded and in what way—positively, negatively, aggressively, with gratitude, a vouch, and so on. We noted that the heuristics we developed are indeed specific to CrimeBB and required some manual effort to develop and refine. It may be that there are some unsupervised methods we can employ in future work to reach the same goal, but for the time being we propose that close inspection remains the best way to get to know your data, remind the reader that the time involved in doing so was not great, and believe that this approach pays dividends in terms of the quality of automated big data labelling.

Another benefit of our models is to try and infer who is talking to whom. It is in the nature of threaded forum conversations that they are not always ordered sequentially: the author of post number 3 in any given thread could be addressing the author of post 1, post 2, or the forum membership as a whole. With our automatically derived labels we can attempt to build a graph of threaded conversations in which each post is a node, and the arcs between nodes may overlap and could be weighted with information such as post type and author intent.

In terms of our understanding of key actors in online forums, these labels are one way to characterise forum members according to their conduct: user *X* asks a lot of questions, responds positively to others and creates many threads, whereas user *Y* makes a lot of comments, writes in a negative fashion, and so on. In other work we have applied the methods discussed here to characterise key actors in HackForums: concretely, we employed NLP techniques to detect whether a user was asking a question or not (Pastrana et al. 2018a). This information was then used to analyse the evolution of expertise and knowledge gathered by key actors across time. We will investigate further applications of forum classification for the characterisation of online behaviour and key actors, including the graph-type analysis discussed above.

Researchers can use NLP techniques such as these to improve their research into underground forums. While we focus on cybercrime, it is possible that similar approaches may also be developed for other types of online forums and marketplaces. These includes online places that are used for expressing extremist ideologies, or trading in other illicit products such as drugs or guns. Similarly, these approaches can be applied to non-English languages. Rather than hand-coding (and translating) millions of posts, by automatically classifying the variables of interest researchers can expand their research scope yet keep costs manageable.

## Conclusion

We consider the use of machine learning and rule-based classifiers to automatically label post type, author intent and addressee in hacking-related online forum data. Of these, author intent is the label type which shows the lowest human agreement and classifier performance. Such is the polysemous and sometimes ambiguous nature of language that it can be difficult to understand and label the intent of another author. More training data, feature engineering and perhaps a deeper semantic analysis of the texts could go some way to addressing this problem. Classifier performance for post type and addressee, on the other hand, are very good, with *F*-measures for each of over 0.8, reflecting the higher inter-annotator agreement and generally less ambiguous nature of these annotation classes.

We discuss the potential applications of these labelled data and note many avenues for future investigation, including further gold-standard annotation and machine learning experimentation, improved pre-processing to better normalise the language found in CrimeBB texts, and the inclusion of such labels in attempts to automatically identify key actors in hacking-related forums.

## Footnotes

1 To be clear on terminology: we refer to a whole website as a forum, on which pages are set aside for discussion of defined topics in bulletin boards, with users participating in conversation threads via individual posts.

2 Namely: Computer and Online Gaming; Cryptography and Encryption Market; Decompiling; Reverse Engineering; Disassembly, and Debugging; Domain Trading; Ebook Bazaar; HF API; Marketplace Discussions; Remote Administration Tools; Secondary Sellers Market; Shopping Deals; Web Browsers; Windows 10; World of Warcraft.

3 Having narrowed down from a larger set of 15, with original labels ‘helpful’, ‘admiration’ and ‘laughter’ being collapsed into the ‘positive’ category, ‘want to have’ merged into ‘requestX’, ‘disapproval’ and ‘sarcasm’ going into ‘negative’, and ‘update’ collapsed into ‘neutral’.

4 See http://datascience.la/benchmarking-random-forest-implementations for random forest speed comparisons.

5 The authors demonstrate with a document classification example that LIBLINEAR can be around 100 times faster than LIBSVM for certain tasks; https://www.csie.ntu.edu.tw/~cjlin/liblinear.
